# Higher satisfaction after total knee arthroplasty using restricted inverse kinematic alignment compared to adjusted mechanical alignment

**DOI:** 10.1007/s00167-020-06165-4

**Published:** 2020-07-31

**Authors:** Philip Winnock de Grave, Thomas Luyckx, Kurt Claeys, Thomas Tampere, Jonas Kellens, Jacobus Müller, Paul Gunst

**Affiliations:** 1 Dept. Orthopaedic Surgery, AZ Delta Roeselare, Brugsesteenweg 90, 8800 Roeselare, Belgium; 2 Dept. Orthopaedic Surgery, UZ Leuven, Herestraat 49, 3000 Leuven, Belgium; 3 Dept. Rehabilitation Sciences, KU Leuven, Spoorwegstraat 12, 8200 Brugge, Belgium; 4 Dept. Orthopaedic Surgery, UZ Gent, De Pintelaan 185, 9000 Gent, Belgium; 5 ReSurg SA, Rue Saint-Jean 22, 1260 Nyon, Switzerland

**Keywords:** Arthroplasty, Knee replacement, Patient-specific alignment, Inverse kinematic alignment, Robotic surgical procedures, Patient-reported outcomes, Patient satisfaction

## Abstract

**Purpose:**

Various alignment philosophies for total knee arthroplasty (TKA) have been described, all striving to achieve excellent long-term implant survival and good functional outcomes. In recent years, in search of higher functionality and patient satisfaction, a shift towards more tailored and patient-specific alignment is seen. The purpose of this study was to describe a restricted ‘inverse kinematic alignment’ (iKA) technique, and to compare clinical outcomes of patients that underwent robotic-assisted TKA performed by restricted iKA vs. adjusted mechanical alignment (aMA).

**Methods:**

The authors reviewed the records of a consecutive series of patients that received robotic-assisted TKA with restricted iKA (*n* = 40) and with aMA (*n* = 40). Oxford Knee Score (OKS) and satisfaction on a visual analogue scale (VAS) were collected at a follow-up of 12 months. Clinical outcomes were assessed according to patient acceptable symptom state (PASS) thresholds, and uni- and multivariable linear regression analyses were performed to determine associations of OKS and satisfaction with six variables (age, sex, body mass index (BMI), preoperative hip–knee–ankle (HKA) angle, preoperative OKS, alignment technique).

**Results:**

The restricted iKA and aMA techniques yielded comparable outcome scores (*p* = 0.069), with OKS, respectively, 44.6 ± 3.5 and 42.2 ± 6.3. VAS Satisfaction was better (*p* = 0.012) with restricted iKA (9.2 ± 0.8) compared to aMA (8.5 ± 1.3). The number of patients that achieved OKS and satisfaction PASS thresholds was significantly higher (*p* = 0.049 and *p* = 0.003, respectively) using restricted iKA (98% and 80%) compared to aMA (85% and 48%). Knees with preoperative varus deformity, achieved significantly (*p* = 0.025) better OKS using restricted iKA (45.4 ± 2.0) compared to aMA (41.4 ± 6.8). Multivariable analyses confirmed better OKS (*β* = 3.1; *p* = 0.007) and satisfaction (*β* = 0.73; *p* = 0.005) with restricted iKA.

**Conclusions:**

The results of this study suggest that restricted iKA and aMA grant comparable clinical outcomes at 12-month follow-up, though a greater proportion of knees operated by restricted iKA achieved the PASS thresholds for OKS and satisfaction. Notably. in knees with preoperative varus deformity, restricted iKA yielded significantly better OKS and satisfaction than aMA.

**Level of evidence:**

Level III, comparative study.

## Introduction

In striving to improve patient satisfaction, alignment in total knee arthroplasty (TKA) is gaining increased attention in recent years. Various alignment philosophies have been described, and can be considered as three main categories [19]: (1) Systematic alignment, such as mechanical alignment (MA) [13] and anatomic alignment (AA) [12], aims for a strict neutral coronal alignment with a hip–knee–ankle (HKA) angle of 180°; (2) Patient-specific alignment, such as kinematic alignment (KA) [11], aims to maintain the native limb alignment; (3) Hybrid alignment, such as adjusted mechanical alignment (aMA) [25] and restricted kinematic alignment (rKA) [2], aim to maintain the native coronal alignment within a HKA angle safe zone of 177° to 183°.

In a recent computed tomography (CT) study of 308 non-arthritic knees, Hirschmann et al. [10] estimated that native limb alignment in men and women, respectively, corresponds to MA in 4% and 6%, to AA in 17% and 18%, and to rKA in 45% and 51%. Although patient-specific alignment techniques would maintain native alignment of all knees, safe ranges for postoperative residual varus or valgus remain unknown [2, 8–10] leading some surgeons to opt for hybrid alignment [18].

The KA technique aims to ‘resurface’ the femur maintaining the native femoral joint line obliquity, with the flexion and extension gaps balanced by adjusting the tibial resection. In some cases, KA involves complex algorithms to balance the flexion and extension gaps [19], which may result in more oblique tibial varus resections that sacrifice medial tibial bone stock. The first author introduced a new technique of restricted ‘inverse kinematic alignment’ (iKA) which aims to ‘resurface’ the tibia with equal medial and lateral resections maintaining the native tibial joint line obliquity, with the flexion and extension gaps balanced by adjusting the femoral resections. This technique could, therefore, avoid tibial over-resection and tibia-related postoperative complications. The purpose of this study was to compare clinical outcomes of patients that underwent robotic-assisted TKA performed by restricted iKA vs. those with aMA. The null hypothesis was that both techniques would yield equivalent clinical scores and patient satisfaction. These findings could be relevant to the ongoing efforts, of improving clinical outcomes while restoring physiological joint line obliquity, using patient-specific alignment techniques.

## Materials and methods

### Patients

**Fig. 1:**
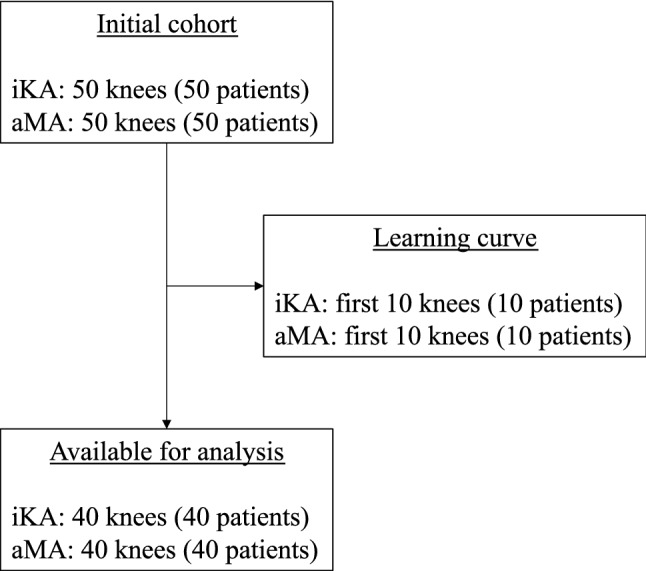
Study flowchart

The authors reviewed the records of a consecutive series of patients that received robotic-assisted TKA (Stryker Triathlon^®^ CR knee) with restricted iKA (*n* = 50, surgeon (1) and with aMA (*n* = 50, surgeon two). To account for the learning curve using robotic-assisted TKA, the first ten cases from each group were excluded (Fig. 1). The indication for surgery was end stage knee osteoarthritis of grade four according to Kellgren–Lawrence classification in at least one of the three knee compartments. Standard radiographic evaluation was carried out on weight-bearing radiographs: anteroposterior, Rosenberg, lateral, and skyline views. Clinical follow-up was organized at 6 weeks, 3 months and 1 year postoperative for both groups. All patients had provided written informed consent for the use of their data and images for research and publishing purposes, and the institutional review board approved the study, according the Helsinki guidelines.

### Surgical technique

All patients underwent general anaesthesia with an additional ultrasound-guided, adductor canal sensory nerve block. For both restricted iKA and aMA groups, exposure was done by a far medial subvastus approach [22] with the medial joint capsule incised in two distinct layers. Arthrotomy was performed far medial just in front of the medial collateral ligament (MCL). When reaching the medial tibia, the longitudinal capsular incision (vertical) was redirected 90° (horizontal) parallel to the tibial plateau in the lateral direction. No soft-tissues were peeled off from the anteromedial tibia. The knee was then brought into flexion and the tourniquet was deflated for the remainder of surgery.

The robotic system (MAKO^®^, Stryker, Kalamazoo, MI, USA) was set up and calibrated following the standard protocol [4]. A preoperative computed tomography (CT) scan of the hip, knee and ankle was uploaded to the TKA application platform for segmentation of a subject-specific three-dimensional model of the knee. During surgery, tibial and femoral trackers were fixed to the bones, and femur and tibia registration was performed by capturing 40 random points on the bony surface of each bone. As it is an image-based system that uses CT, bone registration was made with a sharp probe to guarantee bone contact in areas with cartilage or soft-tissue coverage. An accuracy level of less than 0.5 mm had to be achieved to be able to continue the procedure. All measurements were made intraoperatively using the robotic system user-interface, which has a resolution of 0.5 mm for distances and 0.1° for angles.

**Fig. 2:**
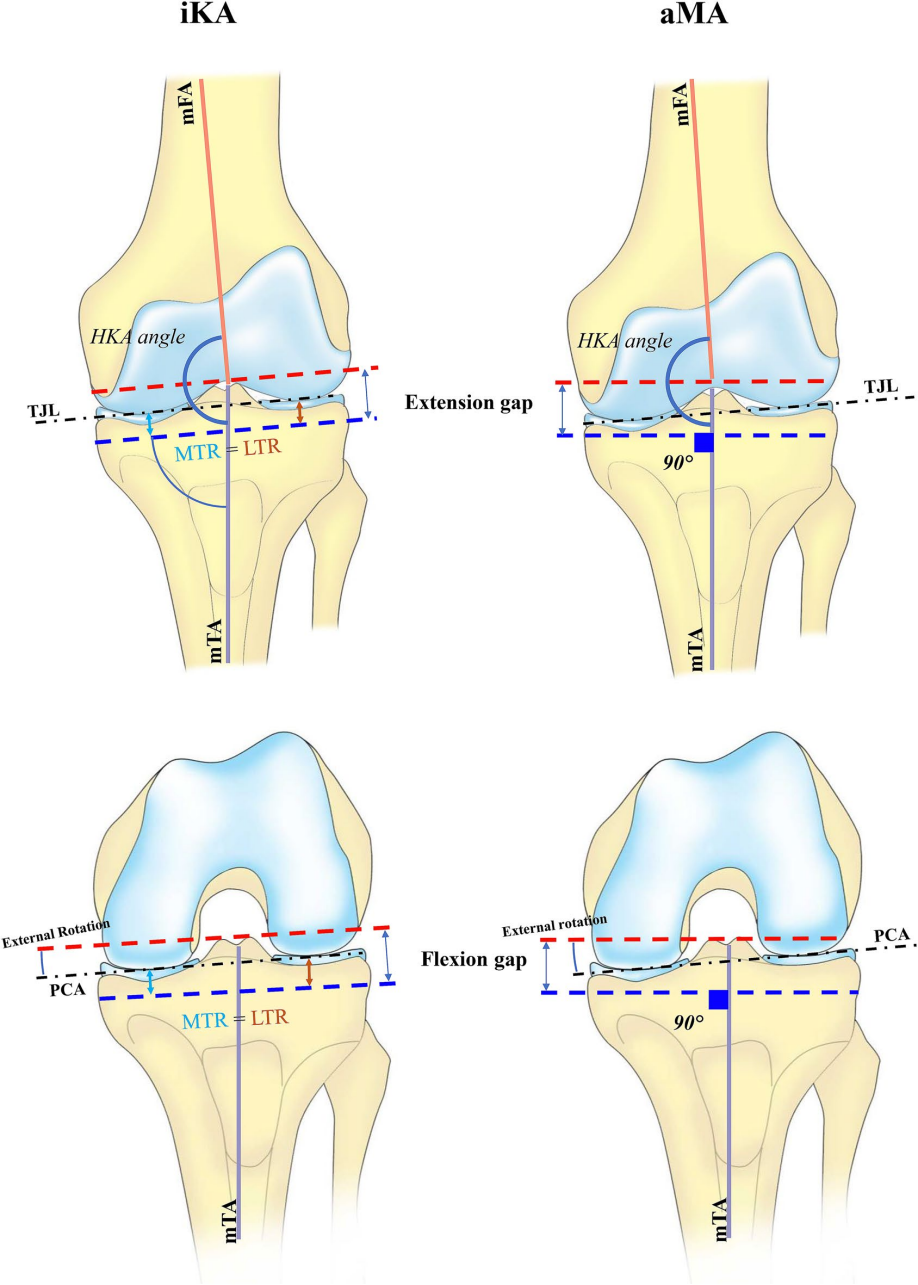
Illustration of the iKA and aMA philosophy in a common knee, with an MPTA of 87°. * **iKA technique:** Tibial resection: tibial resection parallel to tibial joint line *TJL*, medial tibial resection (*MTR)* = lateral tibial resection (*LTR)*. Hip–knee–ankle (*HKA)* angle: restored to pre-arthritic HKA angle by tensioning soft-tissue envelope in extension and performing distal femoral resection. Extension gap laxity: 1–2 mm opening. Flexion gap: femoral rotation governed by soft-tissue tension in flexion with patella-in-place; 1–2 mm medial and 1–3 mm lateral residual laxity.*aMA technique: Tibial resection: 90° with the mechanical tibial axis *mTA*. HKA angle: restored to pre-arthritic HKA angle by soft-tissue envelope in extension and performing distal femoral resection, but within a HKA angle safe zone of 177–183. Extension gap laxity: 1–2 mm opening. Flexion gap: femoral rotation governed by soft-tissue tension in flexion with patella-in-place; 1–2 mm residual laxity medial and lateral. Abbreviations: *mTA* mechanical tibial axis; *mFA* mechanical femoral axis

The navigation tool of the robotic system and a manual ligament tensioning device was used to assess the overall alignment and the soft-tissue envelope both in extension and flexion, with the patella in place. The femoral and tibial components were virtually positioned according to the balancing principles of the alignment technique, i.e., restricted iKA or aMA (Fig. 2).

### iKA

**Fig. 3:**
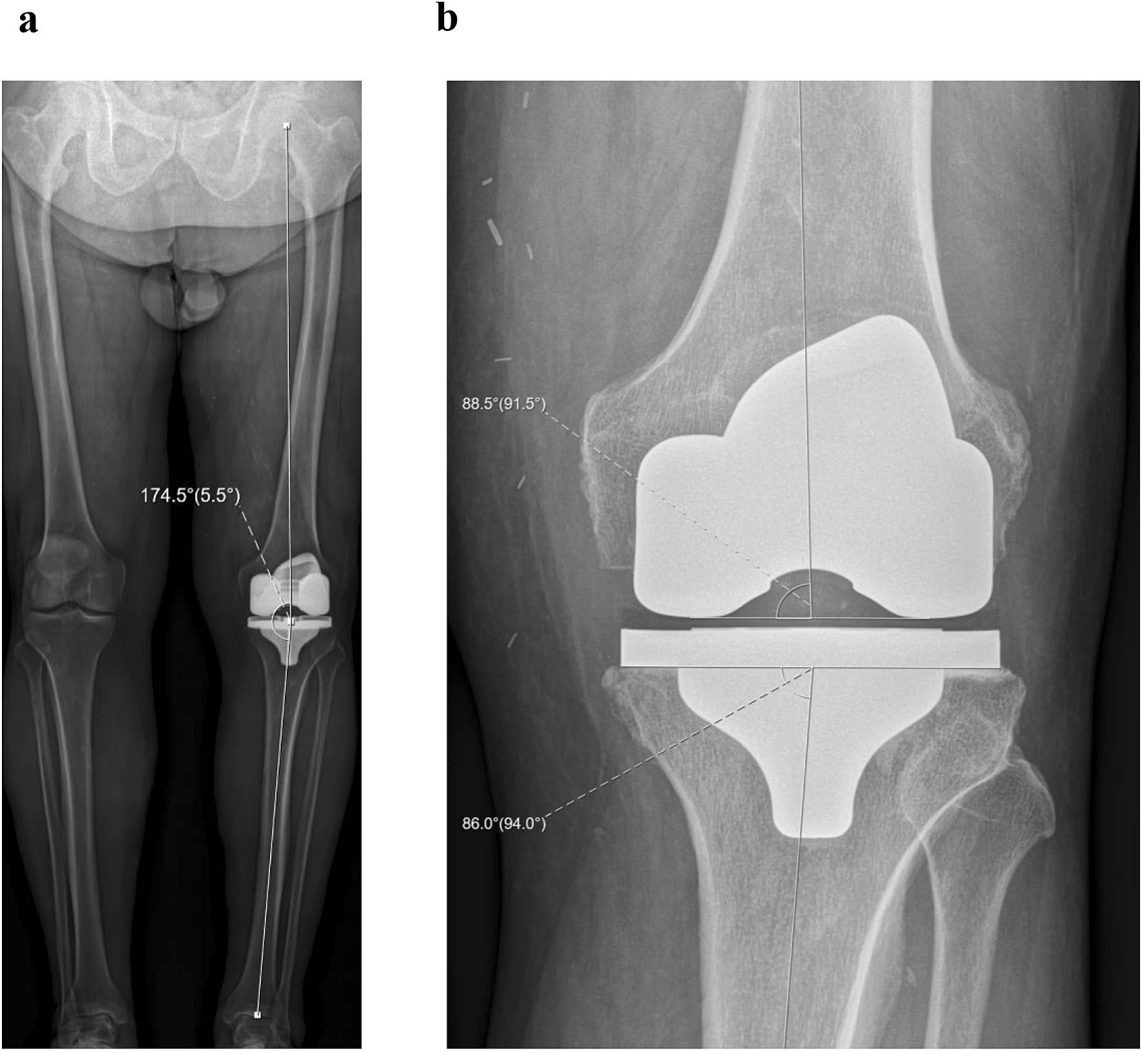
Postoperative radiographs of 76 years, male patient who received a left TKA by iKA. The patient suffered invalidating knee pain caused by tricompartmental osteoarthritis and progressive varus deformity with obliteration of the medial joint space (grade 4). **a** Postoperative standing full-leg X-ray (EOS) showing a restored HKA angle of 174,5° and bilateral symmetrical joint line obliquity, parallel to the floor. **b** Postoperative weight-bearing X-ray of the knee detailing a restored MPTA of 86° incombination with an mLDFA of 91,5°

The tibial component was positioned first by planning a resection of equal amounts of bone medial and lateral on the tibia, taking into account bony wear. The aim was to restore the pre-arthritic medial proximal tibial angle (MPTA), within a safe zone of 84° (varus) to 92° (valgus), which represents native knee alignment in 93% of Caucasian knees [9]. The tibial slope was set equal to the native medial tibial slope. On the femoral side, the femoral component is positioned to restore the medial joint line height both in extension and flexion. The flexion and extension gaps are balanced by adjusting the distal lateral and posterior lateral resection levels on the femur. For the flexion gap, the aim was to achieve residual laxity of 1–2 mm in the medial compartment and 1–3 mm in the lateral compartment. For the extension gap, the aim was to achieve residual laxity of 1–2 mm in both compartments while remaining within an HKA angle safe zone of 174–183°, which accounts for native limb alignment in 96% of Caucasian knees [8] (Fig. 3).

### aMA

The adjusted Mechanical Alignment (aMA) technique is an adaptation of the conventional MA technique but with undercorrection of constitutional coronal deformity, within a limit of ± 3°. The femoral resection is adjusted to preserve mild constitutional deformity and/or reduce more severe deformity while leaving the tibial component mechanically aligned. The tibial component was positioned with the aim to be perpendicular (90°) to the mechanical tibial axis. The tibial slope was equal to the native medial tibial slope. For the flexion gap, the femoral component was positioned to achieve residual laxity of 1–2 mm in both compartments. Likewise, for the extension gap the femoral component was positioned to achieve a residual laxity of 1–2 mm in both compartments while remaining within an HKA angle safe zone of 177–183°.

### Resections

Tibial and femoral resections were done according to the surgeon’s defined intraoperative plan using the haptic robotic-assisted system. As a CT-based navigation tool is used, all planned resection thicknesses are resections of bone, without taking into account the cartilage. The robotic system allowed the surgeon to move the oscillating saw in the defined cutting plane within haptic boundaries, protecting the soft-tissues [4]. The patella was routinely resurfaced using a conventional oscillating saw.

### Rehabilitation

Postoperative rehabilitation protocols included direct mobilization and immediate full weight bearing protected by crutches. With the guidance of a physiotherapist, patients were encouraged to perform exercises with active flexion and active extension movements from day 1. On average, patients stayed three nights in the hospital. Daily physiotherapy sessions were continued at home. The use of crutches was advised during the first 2 weeks. All patients received routine prophylaxis with low-molecular-weight heparin for 4 weeks after surgery. First postoperative appointment at the outpatient clinic was at 6 weeks.

### Clinical scores

Oxford Knee Score (OKS) (worst, 0; best 48) was collected preoperatively and postoperatively at a follow-up of 12 months for all knees. Patients also indicated their satisfaction with the TKA on a Visual Analogue Scale (VAS) (worst, 0; best, 10). Clinical outcomes were assessed according to patient acceptable symptom state (PASS) thresholds. PASS is an absolute threshold proposed for symptomatic variables in osteoarthritis to determine the point beyond which patients consider themselves well and, as such, are satisfied with treatment. Recognized PASS thresholds are 37 for OKS [21] for OKS, and eight for VAS Satisfaction [3].

### Statistical analyses

Descriptive statistics were used to summarize the data. Shapiro–Wilk tests were used to assess the normality of distributions. Differences in means between restricted iKA and aMA knees were, respectively, tested using the student *t* test or analysis of variance, whereas associations between categorical variables were tested using the Kruskal–Wallis test. Based on the classification of Hirschmann et al. [9], which distinguishes five tibial phenotypes, the present study considered only three tibial phenotypes (varus, MPTA < 85°; neutral, 85° ≤ MPTA ≤ 89°; valgus, MPTA > 89°). This simplification was achieved by not considering different extents of varus and valgus alignment, and by defining the thresholds as ± 2° instead of ± 1.5°. Femoral phenotypes were categorised according to their mechanical lateral distal femoral angles (mLDFA) using the classification of Yazdi et al. [26] (varus, mLDFA > 89°; neutral, 85° ≤ mLDFA ≤ 89°; valgus, mLDFA < 85°). Preoperative radiographs were assessed according the Kellgren–Lawrence classification by two investigators involved in this study. Interobserver agreement was calculated using intraclass correlation coefficients (ICC) which can be interpreted as follows [5]: < 0.40 poor; 0.40–0.59 fair; 0.60–0.74 good; 0.75–1.00 excellent. The differences between the pre-, postoperative and net change in OKS, and postoperative VAS Satisfaction in the restricted iKA and aMA groups were analysed in three ways: (1) The difference in means between the two groups; (2) the difference in means between the two groups with knees categorised according to preoperative deformity, i.e., varus (HKA angle < 177°), neutral (HKA angle = 177–183°) and valgus (HKA angle > 183°); and (3) the difference in proportions (%) of knees that were above the PASS thresholds. Considering the findings of Dossett et al. [6] who reported OKS (40 ± 10.2) in knees after KA TKA, and to determine whether a difference of five points in OKS is statistically significant, a priori sample size calculation indicated that a minimum of 39 knees per group was necessary to achieve a power of 70% (G*Power 3.1, Heinrich-Heine-Universität Düsseldorf, Germany). To ascertain detection of the effects of alignment technique on OKS, the sample size used for the present study was adequate, 80 knees with 40 in each group. Uni- and multivariable linear regression analyses were performed to determine associations of three outcomes (Postoperative OKS, OKS net change, VAS satisfaction) and six variables (age, sex, BMI, preoperative HKA angle, preoperative OKS, alignment technique). Multivariable regression models were deemed sufficiently powered, considering the recommendations of Austin and Steyerberg [4] of ten Subject Per Variable (SPV). A *p* value < 0.05 was considered statistically significant. Statistical analyses were performed using R version 3.3.2 (R Foundation for Statistical Computing, Vienna, Austria).

## Results

### Preoperative demographics

The mean age and BMI, and distributions of sex, preoperative HKA angle, MPTA, mLDFA and Kellgren–Lawrence classification were the same between the 2 groups (Table 1). Preoperative HKA angles revealed that most knees were in varus (HKA angle < 177°; restricted iKA, 173.3° ± 1.9° (*n* = 23) vs. aMA, 173.4° ± 2.2° (*n* = 21)), followed by neutral (HKA angle = 177–183°; restricted iKA, 178.7° ± 2.2° (*n* = 13) vs. aMA, 179.7° ± 2.3° (*n* = 15)) and only a few in valgus (HKA angle > 183°; restricted iKA, 185.0° ± 0.8° (*n* = 4) vs. aMA, 185° ± 0.8° (*n* = 4)). Interobserver agreement on Kellgren–Lawrence classifications was excellent (ICC > 0.9).

**Table 1:** Patient demographics

	iKA (*n* = 40)		aMA (*n* = 40)		*p* value
	Mean ± SD (%)	(range)	Mean ± SD (%)	(range)	
**Age** (years)	69.9 ± 8.3	(54–86)	67.4 ± 9.5	(50–89)	*n.s*
**BMI** (kg/m^2^)	29.2 ± 4.8	(21.4–42.6)	30.0 ± 5.3	(21.3–45.1)	*n.s*
**Women**	25 (60%)		23 (58%)		*n.s*
**Preoperative HKA angle**					
Varus knees (< 177°)	23 (58%)		21 (53%)		*n.s*
Neutral knees (177–183°)	13 (33%)		15 (38%)		*n.s*
Valgus knees (> 183°)	4 (10%)		4 (10%)		*n.s*
**Preoperative MPTA**					
Varus knees (< 85°)	1 (3%)		4 (10%)		*n.s*
Neutral knees (85–89°)	38 (95%)		31 (78%)		*n.s*
Valgus knees (> 89°)	1 (3%)		5 (13%)		*n.s*
**Preoperative mLDFA**					
Varus knees (> 89°)	8 (20%)		5 (13%)		*n.s*
Neutral knees (85–89°)	32 (80%)		35 (88%)		*n.s*
Valgus knees (< 85°)	0 (0%)		0 (0%)		
**Kellgren-Lawrence classification**					
Medial compartment					*n.s*
≤ 2	6		8		
3	9		11		
4	25		21		
Lateral compartment					*n.s*
≤ 2	21		19		
3	10		11		
4	9		10		
Patellofemoral compartment					*n.s*
≤ 2	8		3		
3	13		17		
4	19		20		

*iKA* inverse kinematic alignment; *aMA* adjusted mechanical alignment; *SD* standard deviation; *deg*, degrees; BMI, body mass index; *HKA*, Hip-Knee-Ankle; *MPTA* medial proximal tibial angle, *mLDFA* mechanical lateral distal femoral angle

### Overall outcomes

The difference in preoperative MPTA between the two groups was statistically significant (*p* = 0.021), but clinically not meaningful, since the difference was < 1° (Table 2). The postoperative MPTA and HKA angles were significantly (*p* < 0.001 and *p* = 0.003, respectively) more in varus in the restricted iKA group (87.1° ± 1.4° and 178.3° ± 2.1°, respectively), compared with the aMA group (89.6° ± 0.9° and 179° ± 1.9°, respectively). Femoral components were significantly (*p* < 0.001) less externally rotated relative to the native PCA in the restricted iKA group (2.3° ± 1.4°), compared with the aMA group (4.8° ± 2.3°). Medial tibial resections were significantly (*p* < 0.001) deeper in the restricted iKA group (5.4 ± 0.9 mm), compared to the aMA group (4.4 ± 1.2 mm).

**Table 2:** Intraoperative measurements, settings and clinical scores

	iKA (*n* = 40)	aMA (*n* = 40)	*p* value
	Mean ± SD	Mean ± SD	
**Angles**			
HKA angle			
Preoperative (deg)	176.3 ± 4.3	176.9 ± 4.6	*n.s*
Postoperative (deg)	178.3 ± 2.1	179.6 ± 1.9	*0.003*
Net change (deg)	*2.0* ± *2.8*	*2.7* ± *3.2*	*n.s*
MPTA			
Preoperative (deg)	86.7 ± 1.3	87.4 ± 1.7	*0.021*
Postoperative (deg)	87.1 ± 1.4	89.6 ± 0.9	< *0.001*
Net change (deg)	*0.4* ± *0.7*	*2.2* ± *1.5*	< *0.001*
mLDFA			
Preoperative (deg)	88.0 ± 1.4	87.7 ± 1.4	*n.s*
Postoperative (deg)	88.8 ± 1.4	90.0 ± 1.6	< *0.001*
Net change (deg)	*0.9* ± *1.2*	*2.3* ± *1.0*	< *0.001*
Femoral rotation from PCA (deg)	2.3 ± 1.4	4.8 ± 2.3	< *0.001*
Femoral rotation from TEA (deg)	− 0.7 ± 2.1	2.2 ± 2.5	< *0.001*
Tibial Slope (deg)	4.2 ± 1.2	4.1 ± 1.6	*n.s*
**Resections**			
Tibial resection			
Medial (mm)	5.4 ± 0.9	4.4 ± 1.2	< *0.001*
Lateral (mm)	6.1 ± 1.2	6.2 ± 1.3	*n.s*
Femoral resection			
Distal medial (mm)	6.4 ± 0.8	7.1 ± 1.4	*0.021*
Distal lateral (mm)	4.7 ± 1.3	3.4 ± 1.6	< *0.001*
Posterior medial (mm)	8.2 ± 1.2	9.4 ± 1.4	< *0.001*
Posterior lateral (mm)	6.6 ± 1.0	5.7 ± 2.0	*0.014*
**Clinical scores**			
OKS (worst, 0; best, 48)			
Preoperative	26.3 ± 6.4	27.2 ± 5.2	*n.s*
Postoperative	44.8 ± 3.5	42.2 ± 6.3	*n.s*
Net change	*18.6* ± *7.0*	*15.0* ± *8.6*	*n.s*
VAS Satisfaction (worst, 0; best, 10)	9.2 ± 0.8	8.5 ± 1.3	*0.012*
PASS achieved			
OKS	39 (98%)	34 (85%)	*0.049*
VAS satisfaction	32 (80%)	19 (48%)	*0.003*

*iKA*, inverse kinematic alignment; *aMA* adjusted mechanical alignment; *SD* standard deviation; *deg* degrees; *HKA* Hip-Knee-Ankle; *MPTA* medial proximal tibial angle; *mLDFA* mechanical lateral distal femoral angle; *PCA* posterior condylar axis; *TEA* transepicondylar axis; *OKS* Oxford Knee Score; *VAS* visual analog scale; *PASS* patient acceptable symptom state

No complications occurred during surgery. Knee stability and patellar tracking were assessed with no necessity of soft-tissue and lateral releases in both groups. In the aMA group, one patient received a mobilisation under analgesia at 2 months postoperatively. None of the knees were revised or being considered for revision at the latest follow-up.

There were no significant differences in preoperative OKS, postoperative OKS or the net change in OKS between the two groups at 1-year follow-up. VAS satisfaction was significantly (*p* = 0.012) higher in the restricted iKA group (9.2 ± 0.8) compared with the aMA group (8.5 ± 1.3). There were significantly (*p* = 0.049) more knees that exceeded the OKS PASS threshold with restricted iKA [39 (98%)], compared with aMA [34 (85%)]. Likewise, significantly (*p* = 0.003) more knees exceeded the satisfaction PASS threshold using restricted iKA [32 (80%)] compared to using aMA [19 (48%)].

### Effect of preoperative deformity

The alignment, resections and clinical outcomes with restricted iKA and aMA on varus (HKA < 177°), neutral (HKA = 177–183°) and valgus (HKA > 183°) knees are shown in (Table 3). The net change in HKA angle was significantly (*p* < 0.001) less for varus knees in the restricted iKA group (3.8° ± 0.9°), compared with the aMA group (5.2° ± 1.4°). There were no significant differences in postoperative MPTA and HKA of valgus knees between the two groups. Postoperative and net improvement of OKS in varus knees was significantly (*p* = 0.025 and *p* = 0.011, respectively) higher in the restricted iKA group (45.4 ± 2.0 and 19.7 ± 6.4, respectively) compared with the aMA group (41.4 ± 6.8 and 13.7 ± 9.1, respectively). Finally, the VAS satisfaction in varus knees was significantly (*p* = 0.018) higher in the restricted iKA group (9.2 ± 0.8) compared with the aMA group (8.6 ± 0.9).

**Table 3:** Intraoperative measurements, settings and clinical scores for knees categorised according to preoperative HKA angle

	Varus			Neutral			Valgus		
	iKA (*n* = 23)	aMA (*n* = 21)	*p* value	iKA (*n* = 13)	aMA (*n* = 15)	*p* value	iKA (*n* = 4)	aMA (*n* =4)	*p* value
	Mean ± SD	Mean ± SD		Mean ± SD	Mean ± SD		Mean ± SD	Mean ± SD	
**Angles**									
HKA angle									
Preoperative (deg)	173.3 ± 1.9	173.4 ± 2.2	*n.s*	178.7 ± 2.2	179.7 ± 2.3	*n.s*	185.0 ± 0.8	185.0 ± 0.8	*n.s*
Postoperative (deg)	177.1 ± 1.7	178.5 ± 1.8	*0.016*	179.6 ± 1.1	180.3 ± 0.9	*0.030*	180.9 ± 1.0	182.4 ± 1.1	*n.s*
Net change (deg)	*3.8* ± *0.9*	*5.2* ± *1.4*	< *0.001*	*0.9* ± *2.1*	*0.6* ± *1.9*	*n.s*	− *4.1* ± *0.9*	− *2.6* ± *1.3*	*n.s*
MPTA									
Preoperative (deg)	86.2 ± 1.0	86.9 ± 1.4	*0.030*	87.2 ± 1.1	87.9 ± 1.9	*n.s*	88.1 ± 2.1	87.9 ± 2.2	*n.s*
Postoperative (deg)	86.6 ± 1.2	89.3 ± 0.8	< *0.001*	87.6 ± 1.1	89.8 ± 0.9	< *0.001*	88.6 ± 1.6	90.3 ± 0.5	*n.s*
Net change (deg)	0.4 ± 0.6	2.4 ± 1.4	< *0.001*	0.4 ± 0.9	1.9 ± 1.6	*0.006*	0.5 ± 0.7	2.4 ± 1.9	*n.s*
mLDFA									
Preoperative (deg)	88.7 ± 1.0	88.3 ± 1.0	*n.s*	87.2 ± 1.2	87.5 ± 1.4	*n.s*	86.4 ± 1.1	85.6 ± 0.5	*n.s*
Postoperative (deg)	89.5 ± 1.2	90.7 ± 1.5	*0.003*	88.0 ± 1.0	89.5 ± 1.2	*0.003*	87.7 ± 1.2	87.9 ± 0.6	*n.s*
Net change (deg)	0.8 ± 1.5	2.4 ± 0.9	*0.026*	0.9 ± 0.8	2.0 ± 1.2	< *0.001*	1.4 ± 0.5	2.3 ± 0.9	*n.s*
Femoral rotation from PCA (deg)	2.5 ± 1.5	5.2 ± 2.4	< *0.001*	2.4 ± 1.2	4.4 ± 2.2	*0.019*	0.8 ± 1.1	3.9 ± 1.7	*n.s*
Femoral rotation from TEA (deg)	0.2 ± 2.1	2.4 ± 2.4	*0.001*	− 1.9 ± 1.4	1.6 ± 2.8	*0.003*	− 1.7 ± 1.2	2.8 ± 1.6	*0.029*
Tibial Slope (deg)	4.5 ± 1.3	4.2 ± 1.4	*n.s*	3.8 ± 0.7	4.0 ± 1.8	*n.s.* ± 1.0	3.8	4.6 ± 2.0	*n.s*
**Resections**									
Tibial resection									
Medial (mm)	5.1 ± 1.0	4.3 ± 1.3	*0.045*	5.7 ± 0.8	4.5 ± 0.9	*0.002*	5.5 ± 0.7	4.5 ± 1.8	*n.s*
Lateral (mm)	5.8 ± 1.2	6.3 ± 1.3	*n.s*	6.5 ± 1.1	5.9 ± 1.6	*n.s*	6.3 ± 1.8	6.6 ± 0.8	*n.s*
Femoral resection									
Distal medial (mm)	6.4 ± 0.8	7.2 ± 1.4	*n.s*	6.2 ± 0.5	7.1 ± 1.3	*0.026*	7.1 ± 1.4	6.5 ± 1.5	*n.s*
Distal lateral (mm)	5.1 ± 1.3	3.5 ± 1.1	< *0.001*	4.3 ± 1.2	3.8 ± 2.1	*n.s*	4.0 ± 0.4	1.9 ± 0.9	*0.029*
Posterior medial (mm)	8.4 ± 0.9	9.6 ± 1.2	*0.002*	8.2 ± 1.7	8.8 ± 1.4	*n.s*	7.1 ± 0.6	10.1 ± 1.5	*0.029*
Posterior lateral (mm)	6.3 ± 1.0	5.4 ± 1.7	*0.020*	6.8 ± 0.9	5.8 ± 2.5	*n.s*	7.3 ± 0.6	6.5 ± 1.4	*n.s*
**Clinical scores**									
OKS (worst, 0; best, 48)									
Preoperative	25.7 ± 5.9	27.7 ± 4.7	*n.s*	26.0 ± 7.8	25.9 ± 6.3	*n.s*	30.0 ± 2.2	29.8 ± 2.1	*n.s*
Postoperative	45.4 ± 2.0	41.4 ± 6.8	*0.025*	43.6 ± 5.3	42.1 ± 6.1	*n.s*	45.3 ± 2.2	46.5 ± 1.7	*n.s*
Net change	*19.7* ± *6*	*13.7* ± *9*	*0.011*	*17.6* ± *8.6*	*16.3* ± *9.1*	*n.s*	*15.3* ± *3.3*	*16.8* ± *2.9*	*n.s*
VAS Satisfaction (worst, 0; best, 10)	9.2 ± 0.8	8.6 ± 0	*0.018*	9.2 ± 0.8	8.2 ± 1.8	*n.s*	9.0 ± 1.2	9.0 ± 1.2	*n.s*

*iKA* inverse kinematic alignment; *aMA* adjusted mechanical alignment; *SD* standard deviation; *deg* degrees; *HKA* Hip-Knee-Ankle; *MPTA* medial proximal tibial angle; *mLDFA* mechanical lateral distal femoral angle; *PCA* posterior condylar axis; *TEA* transepicondylar axis; *OKS* Oxford Knee Score; *VAS* visual analog scale

Varus < 177°; Neutral, 177–183°; Valgus > 183°

### Regression analysis

Univariable analysis revealed decreasing postoperative OKS with age (*β* = − 0.1; *p* = 0.047) and better OKS with restricted iKA (*β* = 2.6; *p* = 0.023). Multivariable analysis confirmed better postoperative OKS with restricted iKA (*β* = 3.1; *p* = 0.007), but also worse OKS for women (*β* = − 2.4; *p* = 0.045) (Table 4). Univariable analysis revealed less improvement in OKS with age (*β* = − 0.2; *p* = 0.049) and preoperative OKS (*β* =  − 1.0; *p* < 0.001), but better improvement with restricted iKA (*β* = 3.6; *p* = 0.043). Multivariable analysis confirmed less improvement in OKS with higher preoperative OKS (*β* = − 1.1; *p* < 0.001), and better improvement with restricted iKA (*β* = 3.1; *p* = 0.007), but also less improvement for women (*β* = -2.4; *p* = 0.045). Univariable analysis revealed better VAS satisfaction with restricted iKA (*β* = 0.70; *p* = 0.005), which was confirmed with multivariable analysis (*β* = 0.73; *p* = 0.005).

**Table 4:** Uni- and multi-variable regression analyses to identify factors associated with OKS and VAS satisfaction after TKA

	Postoperative OKS (worst, 0; best, 48)				Net change in OKS				VAS Satisfaction (worst, 0; best, 10)			
	Univariable		Multivariable (*n* = 80)		Univariable		Multivariable (*n* = 80)		Univariable		Multivariable (*n* = 80)	
	*β* (95% C.I)	*p* value	*β* (95% C.I)	*p* value	*β* (95% C.I)	*p* value	*β* (95% C.I)	*p* value	*β *(95% C.I)	*p* value	*β* (95% C.I)	*p* value
**Age** (years)	− 0.1 (− 0.3 to − 0.0)	*0.047*	− 0.1 (− 0.2– 0.0)	*n.s*	− 0.2 (− 0.4 to − 0.0)	*0.049*	− 0.1 (− 0.2–0.0)	*n.s*	− 0.01 (− 0.04–0.02)	*n.s*	− 0.01 (− 0.04–0.02)	*n.s*
**BMI** (kg/m^2^)	0.2 (− 0.0–0.4)	*n.s*	0.2 (− 0.1 –0.4)	*n.s*	0.3 (− 0.1 –0.6)	*n.s*	0.2 (− 0.1–0.4)	*n.s*	0.03 (− 0.02 –0.08)	*n.s*	0.03 (− 0.03–0.08)	*n.s*
**Women**	− 2.0 (− 4.3–0.4)	*n.s*	− 2.4 (− 4.7 to − 0.1)	*0.045*	0.2 (− 3.5 –3.9)	*n.s*	− 2.4 (− 4.7 to − 0.1)	*0.045*	− 0.27 (− 0.78– 0.25)	*n.s*	− 0.39 (− 0.91–0.13)	*n.s*
**Preoperative OKS**	0.0 (− 0.3–0.2)	*n.s*	− 0.1 (− 0.3–0.1)	*n.s*	− 1.0 (− 1.3 to − 0.8)	< *0.001*	− 1.1 (− 1.3 to − 0.9)	< *0.001*	− 0.03 (− 0.07–0.01)	*n.s*	− 0.03 (− 0.08–0.01)	*n.s*
**Preoperative HKA angle**												
Varus	REF				REF				REF			
Neutral (deg)	− 0.7 (− 3.2–1.8)	*n.s*	0.2 (− 2.3–2.6)	*n.s*	0.1 (− 3.8–4.0)	*n.s*	0.2 (− 2.3– 2.6)	*n.s*	− 0.27 (− 0.82–0.29)	*n.s*	− 0.14 (− 0.69–0.40)	*n.s*
Valgus (deg)	2.4 (− 1.6–6.3)	*n.s*	2.5 (− 1.3–6.3)	*n.s*	− 0.8 (− 7.0 –5.4)	*n.s*	2.5 (1.3 –6.3)	*n.s*	0.09 (0.79–0.97)	*n.s*	0.19 (− 0.65–1.04)	*n.s*
**iKA**	2.6 (0.4–4.9)	*0.023*	3.1 (0.9–5.3)	*0.007*	3.6 (0.1–7.1)	*0.043*	3.1 (− 0.9–5.3)	*0.007*	0.70 (0.21–1.19)	*0.005*	0.73 (0.23–1.22)	*0.005*

*OKS* Oxford Knee Score; *VAS* visual analog scale; *β* regression coefficient; *C.I*, confidence interval; *REF* reference; *deg* degrees; *BMI* body mass index; *HKA* Hip-Knee-Ankle; *iKA*, inverse kinematic alignment

## Discussion

The most important finding of the present study was that robotic-assisted TKA performed by restricted iKA and aMA granted comparable clinical outcome, regarding OKS at 12-month follow-up. However, multivariable analyses revealed significantly better postoperative and net improvement in OKS as well as satisfaction for restricted iKA, compared to aMA. Finally, in knees with preoperative varus deformity, restricted iKA yielded significantly better OKS and satisfaction. These findings, therefore, partly refute the null hypothesis.

The clinical outcomes of the restricted iKA and aMA techniques in the present study were equivalent and, in some cases, better than findings of other studies that compared TKA performed by KA and MA. In a randomised control trial (RCT), Young et al. [6] found no significant difference between KA (*n* = 49) and MA (*n* = 50) in OKS (42 ± 6 and 41 ± 6, respectively) at 24-month follow-up. Conversely, an RCT by Dossett et al. [27] revealed a significant (*p* = 0.005) difference between KA (*n* = 44) and MA (*n* = 44) in OKS (40 ± 10.2 and 33 ± 11.1, respectively) at 24-month follow-up. Interestingly, 90% of knees in the latter study were predisposed to varus alignment, for whom KA resulted in better OKS scores. In the present study, restricted iKA resulted in significantly better OKS compared to aMA for knees with varus deformity.

The restricted iKA technique yielded significantly greater proportions of knees (98%) that reached the OKS patient acceptable symptom state (PASS) threshold, compared to aMA (85%). By definition, the MA technique modifies the joint line obliquity in most cases, unlike patient-specific techniques, such as KA and restricted iKA. Interestingly, Nakajima et al. [16] found better functional outcomes in knees with postoperative joint line obliquity ≥ 2° compared to knees with postoperative joint line obliquity < 2°. Moreover, Thienpont et al. [24] revealed that in varus knees the joint line convergence angle (the difference between the tibial joint line and the femoral joint line) is 3° ± 2°. The restricted iKA technique restores the joint line obliquity of the tibia, whereas KA restores the joint line obliquity of the femur. Therefore, in varus knees, the postoperative joint line in restricted iKA will be 3° ± 2° less oblique, compared to KA.

Valgus knees have been shown to have hypoplastic lateral femoral condyles (mLDFA of 85° ± 2.3°) [15] with neutral tibiae (MPTA of 90° ± 1.5°) [10]. KA aims to resurface the femur, which might result in oblique valgus femoral resections. Subsequent balancing on the tibia might require an oblique varus resection on a neutral tibia, which will sacrifice important medial tibial bone stock. Since the restricted iKA technique aims to resurface the tibia, the tibial resection will be neutral to the tibial mechanical axis, with equal medial and lateral tibial resections, thereby avoiding tibial over-resection and possibly tibia-related complications. In addition, multiplanar hypoplasia of the lateral femoral condyle in valgus knees is a risk factor for patellar instability [7], and there is a correlation between valgus alignment, femoral rotation and trochlear dysplasia [20]. Thienpont et al. [23] suggested that valgus knees, therefore, require external rotation to restore the native trochlear groove. In contrast to KA, restricted iKA allows for external rotation of the femoral component, which could favour functional outcomes in valgus knees.

This study has a number of limitations which should be acknowledged. First, restricted iKA was used by one surgeon, and aMA was used by another surgeon, which could introduce bias. However, both are experienced, high-volume surgeons (> 100 robotic-assisted TKAs annually each), where the surgical approach, robotic assistance, TKA implant and postoperative rehabilitation were identical. Second, both restricted iKA and aMA were performed with robotic assistance, and the findings may not apply to TKA cohorts using conventional instruments. Nevertheless, restricted iKA relies on a ‘tibia-first’ approach, which most surgeons are familiar with, and which could be performed with conventional extramedullary tibial cutting-guides. Third, this was a retrospective study with only 12-month follow-up, and longer term outcomes at 24 and 60 months are still to be confirmed. Finally, preoperative MPTA and mLDFA phenotype classifications were not included in the multivariable analyses due to the small sample size, but distributions of preoperative osteoarthritic phenotypes between the two groups were equal.

This comparative study is the first describing the restricted iKA technique and reporting on functional outcomes. The clinical relevance of these findings is that the new technique of restricted iKA, with more physiological joint line obliquity, is a promising alignment strategy that merits further investigation and comparison to other patient-specific alignment techniques.

## Conclusion

The results of this study suggest that restricted iKA and aMA grant comparable clinical outcomes at 12-month follow-up, though a greater proportion of knees operated by restricted iKA achieved the PASS thresholds for OKS and satisfaction. Notably, in knees with preoperative varus deformity, restricted iKA yielded significantly better OKS and satisfaction than aMA.
